# Surgical outcomes of sacrospinous hysteropexy and hysteropreservation for pelvic organ prolapse: a systematic review of randomized controlled trials

**DOI:** 10.3389/fmed.2024.1399247

**Published:** 2024-07-24

**Authors:** Xinyu Xiao, Xia Yu, Litong Yin, Ling Zhang, Dan Feng, Lushuang Zhang, Zhaolin Gong, Qiang Zhang, Yonghong Lin, Li He

**Affiliations:** ^1^Department of Obstetrics and Gynecology, Chengdu Women's and Children's Central Hospital, School of Medicine, University of Electronic Science and Technology of China, Chengdu, China; ^2^Department of Clinical Laboratory, Chengdu Women's and Children's Central Hospital, Sichuan Provincial People's Hospital, School of Medicine, University of Electronic Science and Technology of China, Chengdu, China

**Keywords:** sacrospinous hysteropexy, pelvic organ prolapse, hysteropreservation, urinary tract infection, meta-analysis

## Abstract

**Objective:**

In several randomized controlled trials (RCTs), sacrospinous hysteropexy and other forms of hysteropreservation have been compared. Nevertheless, there is no definitively best treatment. This study summarized RCT evidence for various uterine preservation surgical procedures.

**Methods:**

From each database inception to August 2023, we searched PubMed, Embase, Cochrane Library, and Web of Science for eligible RCTs. A comparison was made between sacrospinous hysteropexy and other hysteropreservation, including vaginal and abdominal surgery. For categorical and continuous variables, relative risks (RRs) and mean differences (MDs) were calculated using random-effects models.

**Results:**

We reviewed a total 1,398 studies and ultimately included five RCTs that met all inclusion criteria. These five studies included a total of 1,372 uterine POP cases all of whom received transvaginal surgery and had a follow-up period for assessment of recurrence from 12 months to 5 years. There were no significant differences between sacrospinous hysteropexy and other hysteropreservation for the incidences of recurrence (RR,1.24; 95% CI, 0.58 to 2.63; *p* = 0.58) or hematoma (RR,0.70; 95% CI, 0.17 to 2.92; *p* = 0.62). Moreover, neither sacrospinous hysteropexy nor hysteropreservation had any significant effect on the risk of mesh exposure (RR,0.34; 95% CI, 0.03 to 4.31; *p* = 0.41), dyspareunia (RR,0.45; 95% CI, 0.13 to1.6; *p* = 0.22), urinary tract infection (RR,0.66; 95% CI, 0.38 to 1.15; *p* = 0.15), bothersome bulge symptoms (RR,0.03; 95% CI, −0.02 to 0.08; *p* = 0.24), operative time (MD, −4.53; 95% CI, −12.08 to 3.01; *p* = 0.24), and blood loss (MD, −25.69; 95% CI, −62.28 to 10.91; *p* = 0.17). However, sacrospinous hysteropexy was associated with a lower probability of pain (RR,4.8; 95% CI, 0.79 to 29.26; *p* = 0.09) compared with other hysteropreservation.

**Conclusion:**

There was no difference between sacrospinous hysteropexy and hysteropreservation in terms of recurrence, hematoma, mesh exposure, dyspareunia, urinary tract infection, bothersome bulge symptoms, operative time, pain, and blood loss.

**Systematic Review Registration:**

PROSPERO [CRD42023470025].

## Introduction

Female pelvic organ prolapse (POP) occurs when pelvic organs including the vaginal vault, uterus, and bladder, descend into or through the vagina (1). More than 300,000 POP surgeries are performed annually in the United States alone, and 12–19% of women will undergo surgery for POP during their lifetime (2, 3). Hysterectomy is usually performed for POP repair. An increasing number of women desire to preserve the uterus and therefore reproductive function, thus hysterectomy may not provide any benefits (4). Various surgical methods are available for preserving the uterus, so we need to compare the advantages and disadvantages of each.

According to the Chinese Diagnosis and Treatment Guidelines for Pelvic Organ Prolapse (2020 Edition), pelvic floor reconstruction is feasible for patients with no history of uterine disease, no signs of uterine disease on preoperative imaging examination, and who desire uterine preservation. In several countries, sacrospinous hysteropexy is the most commonly used uterus-preserving technique for women undergoing their first POP operation (5). Although sacrospinous hysteropexy is recommended to be completed through the vagina, but there are also studies some cases in which that have it can be successfully completed it through the abdomen. Sacrospinous hysteropexy is the most studied technique for uterus preservation, and favorable results have been demonstrated, although most studies are limited by selection and information bias, short follow-up periods and a lack of adequate control groups (6).

Many surgical repair techniques have been introduced for POP, and the treatment effects vary, which continues to be a challenge in clinical practice. Surgical methods that preserve the uterus, such as Manchester surgery, ischia spinous fascia fixation, uterosacral ligament suspension and transvaginal mesh, have been widely applied in clinical practice. The current practice in the Netherlands for all stages of uterine prolapse is uterus sparing surgery, and 60% of gynecologists prefer sacrospinous hysteropexy over the Manchester procedure as the first choice for the primary treatment of uterine descent (7). However, the use of vaginal mesh for POP remains controversial, as mesh exposure increases the risk for potential complications, and other novel treatment options are being studied (8, 9). These results are based on prospective, nonrandomized, and retrospective cohort studies, thus the differences between sacrospinous hysteropexy and other hysteropreservation techniques may be over-or underestimated.

Several RCTs compared the efficacy and safety of sacrospinous hysteropexy and other preservation techniques in women with POP; however, inconsistent results have been obtained (5, 10–13). Hence, it is important to determine the optimal surgical procedure for women with POP. Therefore, we conducted a systematic review and meta-analysis of RCTs to compare the efficacy and safety of sacrospinous hysteropexy and other hysteropreservation methods in women with POP.

## Methods

### Search strategy

From each database inception to August 2023, we searched PubMed, Embase, Cochrane Library, and Web of Science for eligible RCTs, using keywords such as “prolapse” together with “prolapses” or “uterine preservation surgery” or “sacrospinous hysteropexy” or “hysteropreservation” and “randomized” or “randomly” or “random” without restrictions on language. Only RCTs were eligible. Additionally, to identify ongoing or unpublished trials, we searched ClinicalTrials.gov.

### Inclusion and exclusion criteria

We included eligible studies according to the following criteria:

1. Population: All women with POP symptoms who signed informed consent forms were included.
2. Interventions: The participants in the experimental group underwent sacrospinous hysteropexy via the vagina or abdomen.
3. Control group: The participants in the control group underwent other surgical methods to preserve the uterus via the vagina or abdomen.
4. Outcome: The trials included at least one of the following results: recurrence (apical descent greater than one-third of total vaginal length or anterior or posterior vaginal wall beyond the hymen or retreatment for prolapse, or bothersome bulge symptoms), bleeding volume, infection, pain, surgical time, hematoma, abscess, mesh exposure, dyspareunia, risk exposure, urinary tract infection, or operative time.
5. Study type: Only RCTs were included.

We excluded duplicated literature, reviews, conference abstracts, animal studies, irrelevant articles and non-RCTs. Studies that did not have complete data were also excluded. However, research on mesh techniques were not ruled out.

### Study selection procedure

After removing duplications, two authors independently reviewed the titles and abstracts to exclude any unqualified studies. Then, the full text of the article was extracted and independently screened by two authors. If there was a discrepancy between the two authors, a third author reviewed the controversial information and discussed its inclusion to reach a consensus.

### Assessment of risk of bias

To evaluate the methodological quality of the eligible studies, two authors independently used the Cochrane Risk of Bias tool to assess the risk of bias for the included RCTs, and the risk was assessed as low, high or unclear. Disagreements were discussed and resolved in cooperation with the third author. To ensure the objectivity of our assessment of research risks, we concealed journal titles from the investigators.

### Data extraction

Two authors independently scanned the full texts of all included RCTs and stored the necessary information for each trial, including article details (first author name, publication year, country), study information (study location, number of participants, and baseline information), intervention details (surgical method selection), and outcome information (measurement tools and raw data). To ensure the accuracy of the information, a third reviewer subsequently validated the extracted data and resolved any differences through discussion.

### Statistical analysis

We performed this meta-analysis using Review Manager version 5.3 (Cochrane Collaboration, Oxford). Except for the operative time and estimated blood loss volume which were regarded as continuous variables, the other outcomes were considered dichotomous variables. The categorical and continuous outcomes were assessed using the relative risk (RR) ratio or mean difference (MD) with 95% confidence intervals (CIs) (14). For the pooled estimates, a 95% confidence interval (CI) was calculated. A *p* value < 0.05 was considered to indicate statistical significance. The I^2^ statistic was used to assess the statistical heterogeneity (15), with an I^2^ > 50% indicating significant heterogeneity. Considering the heterogeneity among different studies, a random-effects model was used instead of a fixed-effects model. Publication bias was evaluated by funnel plots (Supplementary Figure 1).

## Results

### Eligible studies and their characteristics

**Figure 1:**
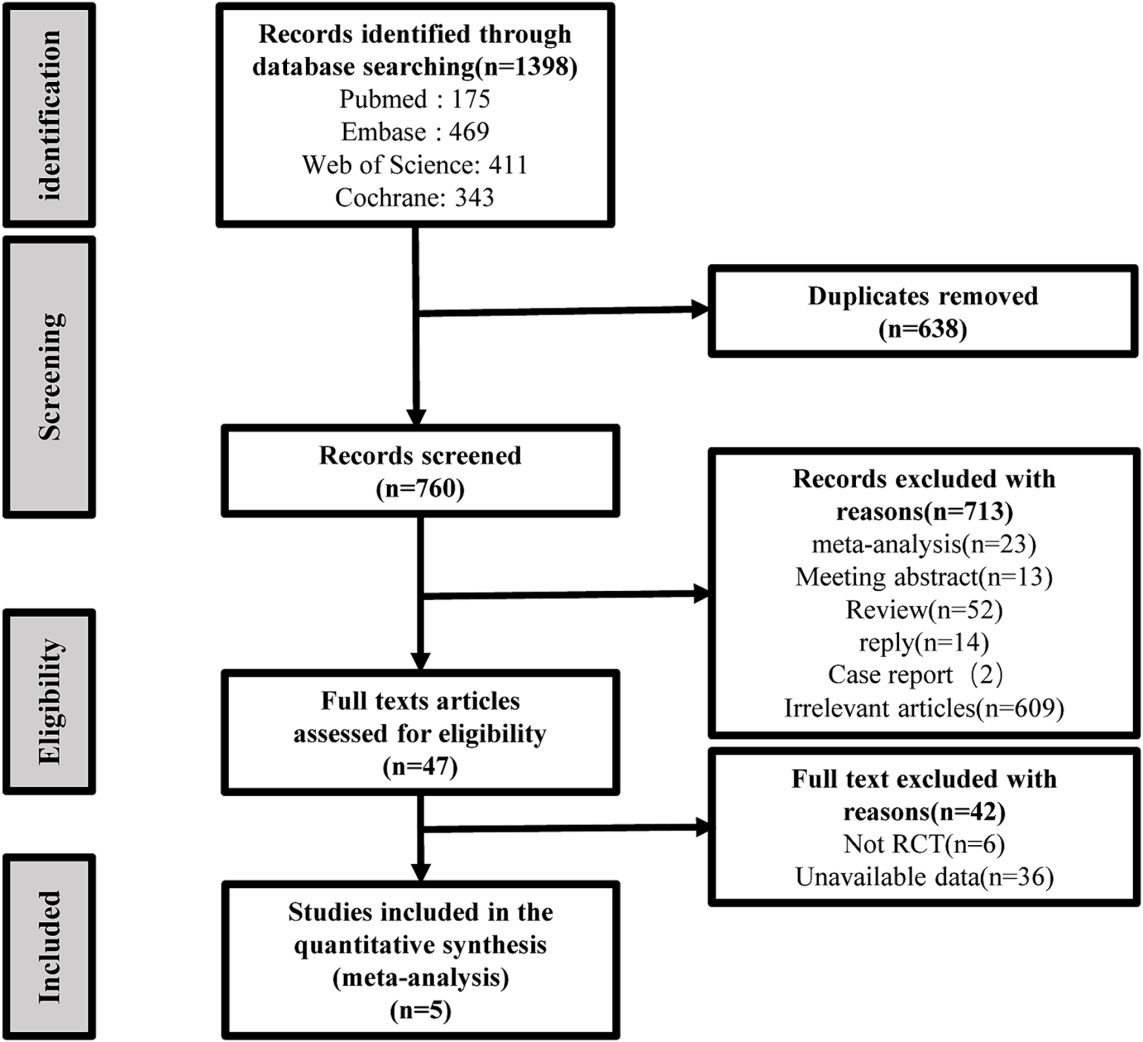
Flow chart of the identification and inclusion of study selection based on the preferred reporting items for systematic reviews and meta-analysis statement.

A total of 1,398 studies were identified through an electronic search, and 760 studies were retained after excluding duplicate articles. In addition, 713 articles were further excluded due to their irrelevant titles and abstracts. A total of 47 studies were searched for further full-text evaluation, and 42 studies were excluded due to a lack of randomized controlled trials (*n* = 6) or complete data (*n* = 36). Finally, 5 randomized controlled trials (including 1,372 women with uterine prolapse) were selected for the final meta-analysis. All the studies included involved transvaginal surgeries. No current eligible randomized controlled trials were found when evaluating the reference lists of relevant reviews and original studies. The details of the literature search and research selection are shown in Figure 1.

The baseline characteristics of the included studies and patients are summarized in Table 1. The included studies were published between 2011 and 2023, and each trial included 22–434 patients. All studies compared sacrospinous hysteropexy with other uterine preservation procedures for patients with POP. Three experiments were conducted in Europe or the United States, and the remaining two were conducted in the Netherlands and Czech Republic. The average age of the patients ranged from 57.15 to 70.2 years, and the follow-up period ranged from 12 to 60 months.

**Table 1:** The main characteristics of the randomized controlled trials.

Study	Intervention	Control	Sample size (Int/Cont)	Mean age (Int/Cont)	Parity (Int/Cont)	BMI (Int/Cont)	History of POP (Int/Cont)	Inclusion criteria	Follow-up duration
Halaska et al. (10)	Sacrospinous fixation (Transvaginal)	Mesh procedures (Transvaginal)	168 (83/85)	64.89 (66.41/63.37)	2.32/2.08	27.62/26.81	NA	POP-Q stage II or greater	12 months
Enklaar et al. (5)	Sacrospinous hysteropexy (Transvaginal)	Manchester procedure (Transvaginal)	434 (217/217)	62.0 (61.0/63.0)	3.0/3.0	25.4/25.2	83.0/77.0	Aged 18 years or older planning to undergo a first surgery for symptomatic pelvic organ prolapse in any stage	2 years
Jelovsek et al. (13)	Sacrospinous ligament fixation (SSLF; Transvaginal)	Uterosacral ligament suspension (ULS; Transvaginal and mesh)	374 (186/188)	57.15 (56.9/57.4)	2.8/3.4	29.2/28.5	12.0/6.0	POP-Q stage II-IV	5 years
Barber et al. (12)	Sacrospinous ligament fixation (SSLF; Transvaginal)	Uterosacral ligament suspension (ULS; Transvaginal and mesh)	374 (186/188)	57.25 (57.2/57.3)	2.0/3.0	29.0/28.7	17.0/9.0	Aged at least 18 years undergoing vaginal surgery for stage II-IV prolapse	2 years
Heinonen et al. (11)	Anterior vaginal wall mesh augmentation with concomitant sacrospinous ligament fixation (SSLF; Transvaginal and mesh)	Posterior intravaginal slingplasty (IVS; Transvaginal and mesh)	22 (8/14)	70.2 (68.0/73.0)	2.0/2.0	24.0/27.0	3.0/4.032(16/16)	Patients with symptomatic vaginal vault prolapse or uterine procidentia	3 years

### Risk of bias in included studies

**Figure 2:**
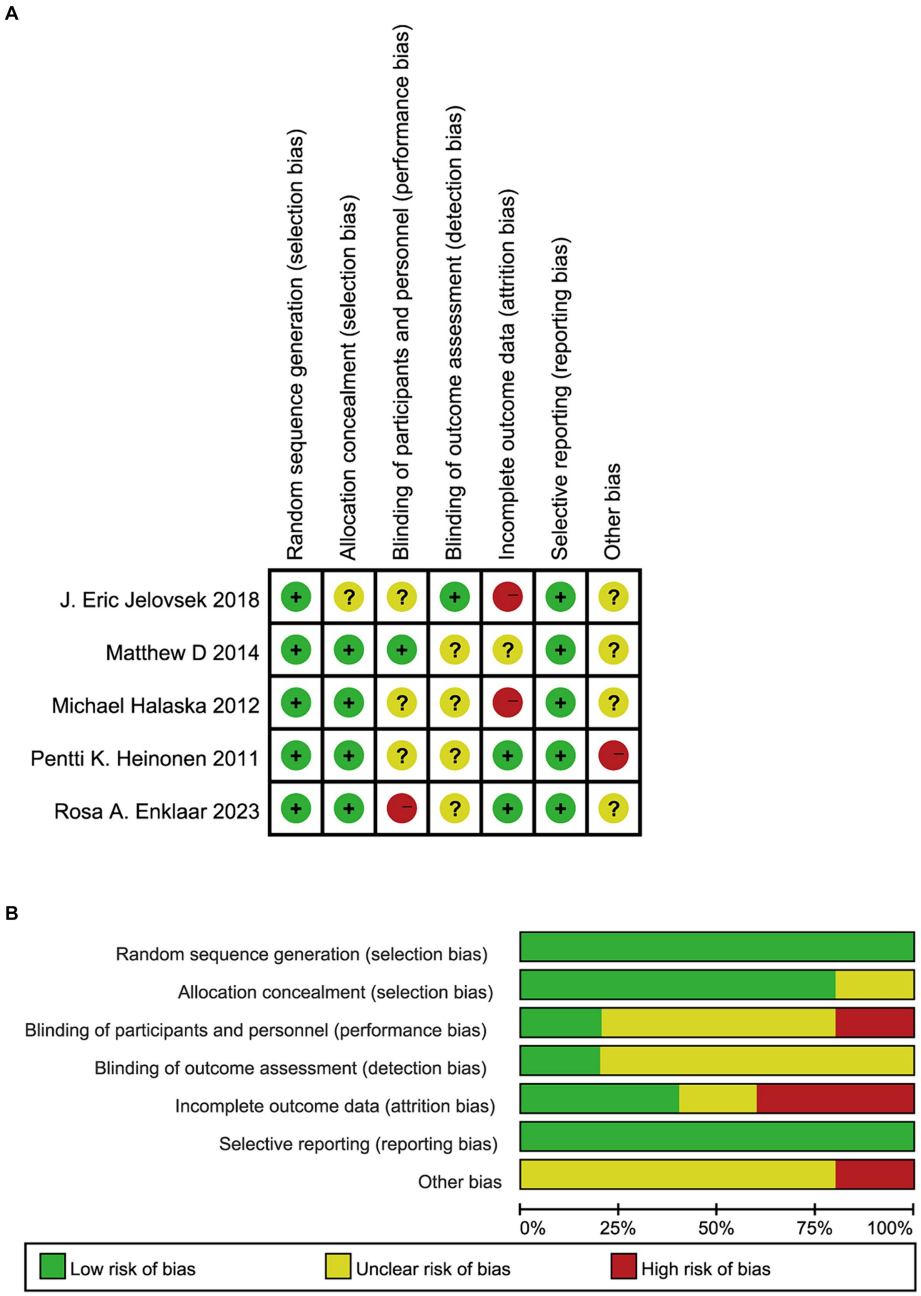
A summary of the results of risk of bias in include RCTs. Green represents a low risk of bias, yellow represents an ambiguous risk of bias, and red represents a high risk of prejudice. The figure **(A)** (deviation risk chart) shows the overall deviation risk for each area. For example,the length of a green rectangle means the number of studies assessed as low bias risk. The figure **(B)** (deviation risk summary) represents the deviation risk in each area of each study. RCTs, randomized controlled trials.

All eligible studies involved random allocation, and five randomized controlled trials were assessed to have a low risk of generating patterns of random sequences. In addition, four trials were conducted with concealment of the allocation. Because of the nature of the intervention, it was not possible to blind clinicians or participants in all studies, but to avoid performance bias and detection bias, the double-blind method was used in one trial. Regarding the outcome, only two studies were identified as having a low risk (Figures 2A,B).

### Meta-analysis

**Figure 3:**
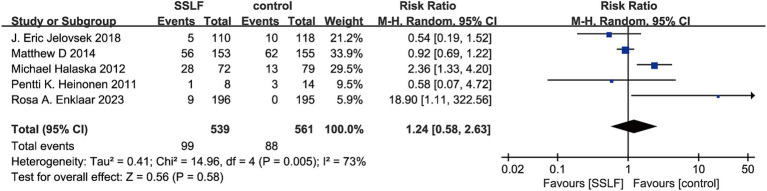
The forest plot of prolapses recurrence. Significant differences were observed between SSLF and control group on prolapse recurrence (*p =* 0.58). CI, confidence interval.

**Figure 4:**
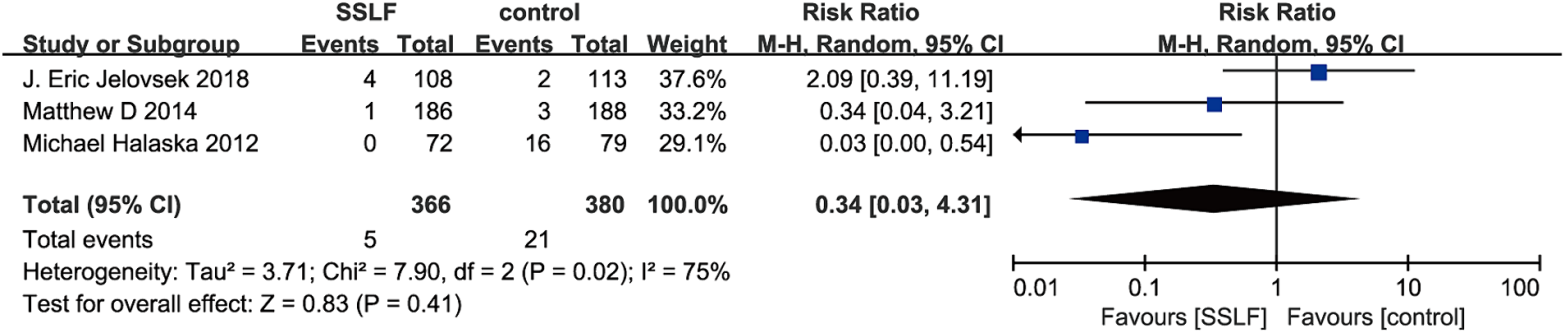
The forest plot of mesh exposure. Significant differences were observed between SSLF and control group on mesh exposure (*p* = 0.41). CI, confidence interval.

There were 5 studies on recurrence and 3 studies on mesh exposure. There was no significant difference in recurrence risk between uterine sacral ligament suspension surgery and other surgical methods (RR, 1.24; 95% CI, 0.58–2.63; *p* = 0.58; Figure 3) or mesh exposure (RR, 0.34; 95% CI, 0.03–4.31; *p* = 0.41; Figure 4). There was significant heterogeneity in the recurrence rate (I^2^ = 73%; *p* = 0.005), and there was also significant heterogeneity in mesh exposure (I^2^ = 75%; *p* = 0.02).

**Figure 5:**
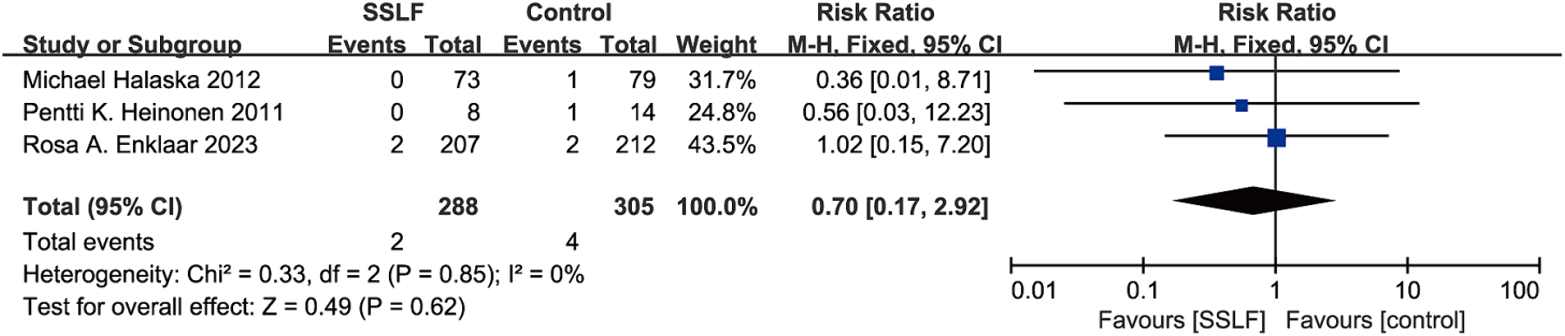
The forest plot of Hematoma. No significant differences were observed between SSLF and control group on Hematoma (*p* = 0.62). CI, confidence interval.

**Figure 6:**
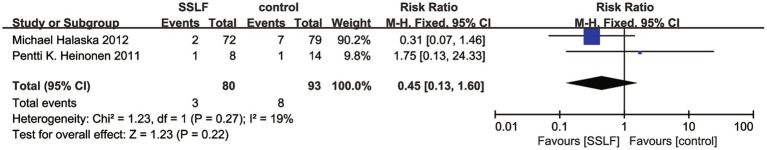
The forest plot of dyspareunia. There was no significant difference in dyspareunia between the SSLF group and the control group (*p* = 0.22). CI, confidence interval.

There were 3 studies on hematoma and 2 studies on dyspareunia. We observed that compared with other surgical methods, uterine sacral ligament suspension surgery had no significant impact on the risk of hematoma (RR, 0.70; 95% CI, 0.17–2.92; *p* = 0.62; Figure 5) or dyspareunia (RR, 0.45; 95% CI, 0.13–1.60; *p* = 0.22; Figure 6) compared to other surgical methods. Hematomas had insignificant heterogeneity (I^2^ = 0%; *p* = 0.85) and dyspareunia have moderate heterogeneity (I^2^ = 19%; *p* = 0.27).

**Figure 7:**
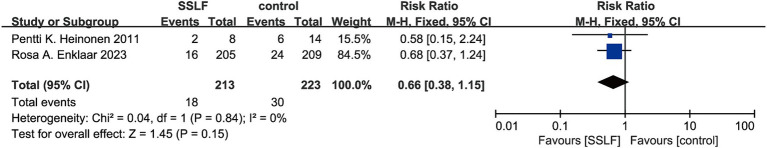
The forest plot of urinary tract infection. No significant differences were observed between SSLF and control group on urinary tract infection (*p* = 0.15). CI, confidence interval.

**Figure 8:**
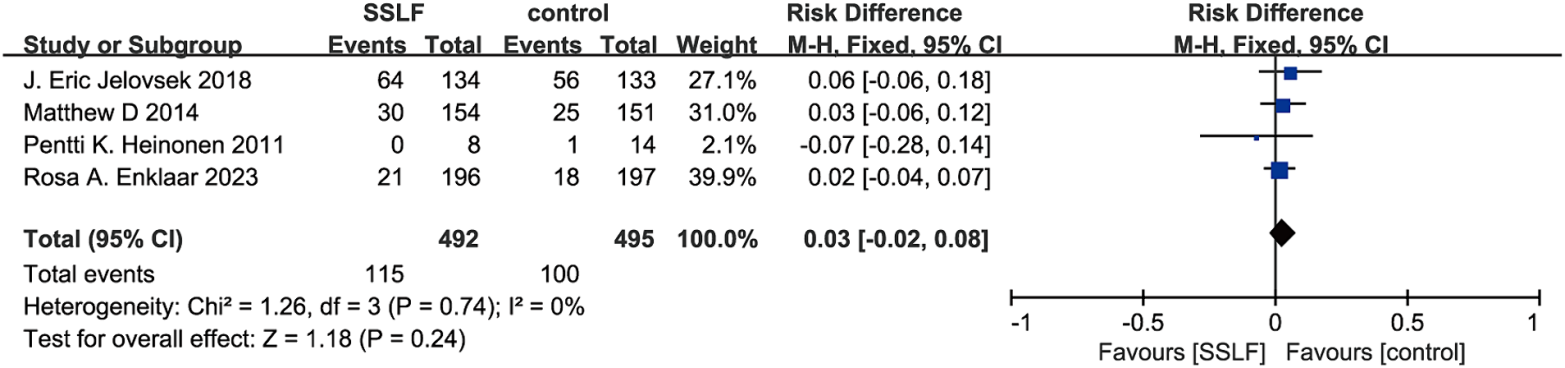
The forest plot of bothersome bulge symptoms. No significant differences were observed between SSLF and control group on bothersome bulge symptoms (*p* = 0.24). CI, confidence interval.

**Figure 9:**
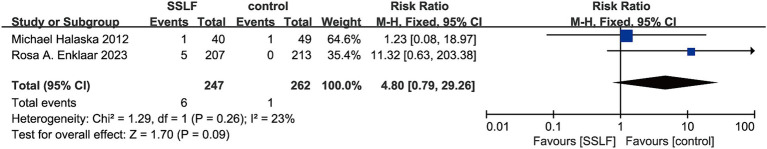
The forest plot of pain. No significant differences were observed between SSLF and control group on pain (*p* = 0.09). CI, confidence interval.

There were 2, 4, and 2 studies on urinary tract infection, bothersome bulge symptoms, and pain, respectively. There was no significant difference in the risk of intraoperative urinary tract infections between sacrospinous hysteropexy and other surgical methods (RR, 0.66; 95% CI, 0.38–1.15; *p* = 0.15; Figure 7), bothersome bulge symptoms (RR, 0.03; 95% CI, −0.02-0.08; *p* = 0.24; Figure 8), or pain (RR, 4.80; 95% CI, 0.79–29.26; *p* = 0.09; Figure 9). In addition, there was nonsignificant heterogeneity in urinary tract infection (I^2^ = 0%; *p* = 0.84), bothersome bulge symptoms (I^2^ = 0%; *p* = 0.74), and pain (I^2^ = 23%; *p* = 0.26).

**Figure 10:**
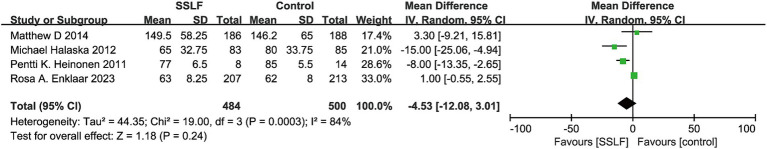
The forest plot of operative time(min). Significant differences were observed between estrogen and control group on operative time (min; *p* = 0.24). CI, confidence interval; MD, mean difference.

**Figure 11:**
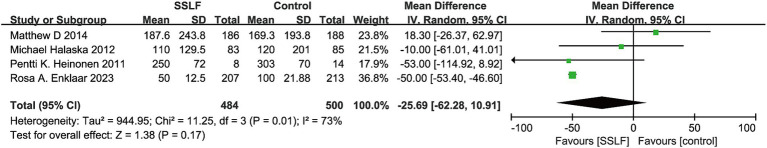
The forest plot of estimated blood loss(ml). Significant differences were observed between estrogen and control group on estimated blood loss (ml; *p* = 0.17). CI, confidence interval; MD, mean difference.

There was 4 and 4 studies on operative time and bleeding volume, respectively. We observed no significant differences in operative time (MD, −4.53; 95% CI, −12.08-3.01; *p* = 0.24; Figure 10) or bleeding volume (MD, −25.69; 95% CI, −62.28-10.91; *p* = 0.17; Figure 11) between the SSLF group and the other surgical methods. In addition, there was significant heterogeneity in surgical time (I^2^ = 84%; *p* = 0.0003) and intraoperative bleeding volume (I^2^ = 73%; *p* = 0.01).

## Discussion

The current systematic review and meta-analysis were based on RCTs and compared the treatment effectiveness of sacrospinous hysteropexy with that of other hysteropreservation techniques in women with POP. This study included 1,372 patients from 5 RCTs, covering a wide range of features. The results of this study indicated that there was no significant difference in recurrence rate, hematoma rate, mesh exposure rate, dyspareunia rate, urinary tract infection rate, pain, bothersome bulge symptoms, surgical time, or risk of bleeding between sacral spine uterine fixation surgery and other uterine preservation procedures.

Currently, no optimal surgical treatment has been identified for correcting POP. Moreover, there is currently a lack of meta-analyses on uterine preservation procedures, and few RCTs have been performed to date. There is a lack of comprehensive evidence indicating whether this technique is optimal. It can only be discussed through some prospective or retrospective studies Therefore, only patients who underwent uterosacral ligament suspension surgery, transvaginal mesh surgery or Manchester surgery were included in the control group after the inclusion criteria were met.

Sacrificial ligament fixation and uterosacral ligament suspension are the two most common and widely studied vaginal root tip surgeries involving the use of native vaginal tissue (16). In this study, we also included relevant RCTs, but no differences were observed in indicators such as recurrence or distressing swelling. This finding is consistent with the risk of uterosacral ligament suspension and sacral spine fixation mentioned in the guidelines written by Geoffrion (17). However, according to these guidelines, uterosacral ligament suspension is associated with a greater risk of ureteral injury, and sacrificial ligament fixation is associated with mild pain in the short term. Shah (18) conducted a multicenter retrospective cohort study consisting of 9,681 women, with a maximum follow-up time of 14.8 years. The incidence of reoperation was lower for uterosacral ligament suspension surgery (9 cases per 1,000 patient years) and sacrificial ligament fixation surgery (13.9 cases per 1,000 patient years). Moreover, 9.3% of patients (43/464) who underwent sacrificial ligament fixation used uterine support after the operation. Jelovsek et al. (13) reported no significant difference in surgical failure rates between USLS and SSLF after 5 years (61.5% for uterosacral ligament suspension and 70.3% for sacrificial ligament fixation) in the eOPTIMAL trial of extended surgery and pelvic muscle training for root apex support deficiency. Barber et al. (19) reported that the postoperative pain patterns associated with uterosacral ligament suspension surgery and sacrificial ligament fixation were similar, with less postoperative pain in both groups of patients. In addition, in Sérgio Brasileiro Martins’ study (20), there was no significant difference in the incidence of urinary tract infection between the sacrificial ligament fixation group and the uterosacral ligament suspension group.

No significant differences were observed in the terms of mesh exposure, or recurrence rates, in this study. In Tyler L Overlolt’s study (21), it was shown that compared to sacrospinous hysteropexy, mesh augmentation surgery did not have any additional benefits as there was no statistically significant difference in the incidence of complications between the two groups (*p* = 0.752). At the last follow-up exam, ofno women in the patch enhanced repair group, experienced patch related complications, including chronic pain or patch exposure. This is consistent with the results of this study. The FDA banned the use of mesh in 2019, thereby leading to a significant decrease in its the use of mesh has significantly decreased in all countries (22). Advances in technology and materials science are likely to influence changes to mesh.

Sacrospinous hysteropexy is one of the most used commonly uterus-sparing techniques, and the Manchester procedure is one of the oldest procedures and is not widely utilized in current clinical practice. During a 10-year follow-up, Jha et al. reported extensive practical changes and an increase of 10% in the number of uterine preservation surgeries performed for uterine descent, including sacrospinous hysteropexy and modified Manchester (23–25). Although modified Manchester and sacral spine uterine fixation are the most common preservation surgeries for uterine descent, obstetricians and gynecologists know little about their preferenceshave rarely expressed a preference for either of these two types of surgeries. In addition to Dutch gynecologists (7), most doctors express concern about the high POP recurrence rate after sacral spine uterine fixation surgery, and the quality of the uterosacral ligament is related to the chance of recurrence. Some doctors select the surgical method based on the patient’s preference. The preference for one of the uterine protection interventions is mainly based on the experience and background of the gynecologists themselves. The lack of information on these two types of uterine preservation procedures hinders evidence-based decision-making, which explains the differences in practical models. Brunes (26) reported that compared with other uterine prolapse surgery methods, Manchester surgery is associated with lower rates of POP, recurrence, symptom recurrence and lower surgical incidence rate. However, the data in the study by Rosa A. Enklaar’s (5), showed that at less than 6 weeks, the number of urinary tract infections in the Manchester group was significantly greater than that in the Sacrospinous hysteropexy group. In summary, group. In summary, additional research is needed to improve evidence-based consultation and collaborative decision-making regarding program selection.

In addition, there have been articles analyzing laparoscopic sacral spine uterine fixation and transvaginal sacral spine uterine fixation. Van IJsselmuiden et al. (27) conducted a multicenter randomized controlled, unblinded, and noninferiority trial in the Netherlands, and reported that laparoscopic sacral intrauterine fixation had a rate of apical septal surgery failure similar to that of transvaginal sacral intrauterine fixation the 12-month. After laparoscopic sacral uterine fixation surgery, overactive bladder and fecal incontinence are more common, but pain is less common. They believe that both have equally good short-term prognoses. Van Oudheusden et al. (28) performed a retrospective study of patients who underwent laparoscopic sacrohysteropexy and vaginal sacrospinous hysteropexy. They found no clinically relevant differences in the success rate (*p* = 0.073), apical septal anatomical failure rate (*p* = 0.711), incidence of vaginal protrusion symptoms (*p* = 0.126), or patient satisfaction (*p* = 0.741), between the two, while the LSH group had longer surgery times and hospital stays. Ronsini et al. (29) conducted a meta-analysis of uterosacral ligament suspension in transvaginal and laparoscopic procedures, but the results did not show that one was superior to the other. However, Douligeris’s meta-analysis (30) on transvaginal and laparoscopic surgery, revealed a potential reduction in the incidence of ureteral damage associated with laparoscopic surgery (OR, 0.19; 95% CI 0.04–0.89; *p* = 0.04) and a seemingly low objective (OR 0.47; 95% CI 0.23–0.97; *p* = 0.04) and subjective recurrence rate (OR 0.46; 95% CI 0.23–0.92; *p* = 0.03). As there is currently no consensus on the optimal surgical procedure, more meaningful clinical studies are needed.

Several potential limitations in our study should be considered. First, although the analysis was based on published randomized controlled trials, the quality of the included studies varied. Second, the included data are relatively limited, and the relevant techniques used are also limited. Third, although our analysis included only randomized controlled trials, the small number of trials included resulted in lower reliability of the results. Fourth, as the analysis is based on published articles, publication bias was inevitable. Therefore, more large-scale, high-quality, randomized, double-blind, placebo-controlled trials are needed in the future to obtain additional evidence for this field of study.

In conclusion, the purpose of this study was to compare the outcomes of sacrospinous hysteropexy with those of other uterine preservation surgeries, and this study suggested that there are no differences in indicators such as recurrence, hematoma, pain, or difficulty during sexual intercourse between sacral spinous hysterectomy and other uterine preservation surgeries. Large-scale randomized studies are crucial for determining the relative advantages of various uterine preservation procedures more clearly.

## Data availability statement

The original contributions presented in the study are included in the article/Supplementary material, further inquiries can be directed to the corresponding authors.

## Author contributions

XX: Writing – original draft. XY: Writing – original draft, Funding acquisition. LY: Writing – original draft. LiZ: Data curation, Writing – original draft. DF: Writing – original draft. LuZ: Data curation, Writing – original draft. ZG: Writing – original draft. QZ: Writing – original draft. YL: Project administration, Writing – review & editing, Funding acquisition. LH: Project administration, Writing – review & editing, Funding acquisition.
