# Supplementary figures and images for: Surgical outcomes of sacrospinous hysteropexy and hysteropreservation for pelvic organ prolapse: a systematic review of randomized controlled trials
Source: Front Med (Lausanne). 2024 Jul 24;11:1399247. doi: 10.3389/fmed.2024.1399247 (PMC11303157; doi:10.3389/fmed.2024.1399247)

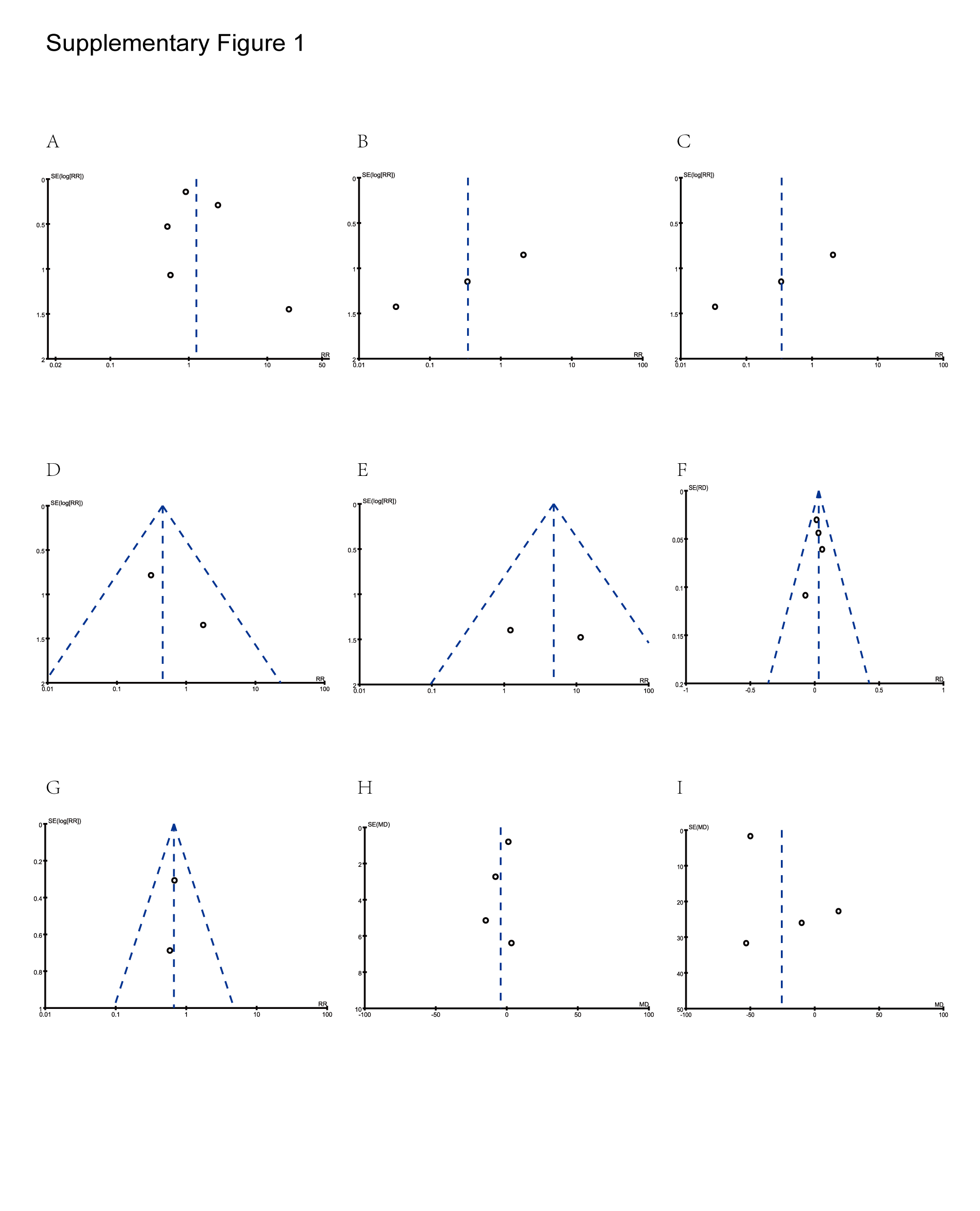

Supplement: Supplementary file 1 [file Image_1.TIF]
